# Editorial: Trait Emotional Intelligence: Foundations, Assessment, and Education

**DOI:** 10.3389/fpsyg.2020.00608

**Published:** 2020-04-16

**Authors:** Juan-Carlos Pérez-González, Donald H. Saklofske, Stella Mavroveli

**Affiliations:** ^1^Emotional Education Laboratory (EDUEMO Lab), Faculty of Education, National University of Distance Education (UNED), Madrid, Spain; ^2^Department of Psychology, University of Western Ontario, London, Canada; ^3^Department of Surgery and Cancer, Imperial College London, London, United Kingdom

**Keywords:** trait emotional intelligence, emotional competence, emotional intelligence, trait emotional self-efficacy, emotions, emotional education

## Introduction

Trait Emotional Intelligence (Trait EI) theory was introduced by Petrides in 2001 and “describes our perceptions of our emotional world: what our emotional dispositions are and how good we believe we are in terms of perceiving, understanding, managing, and utilizing our own and other people's emotions” (Petrides et al., 2018, p. 50). Although there are still different conceptualizations, models, and measures (self-reports or other-reports) of EI, so far only the Trait EI theory (see Petrides et al., 2007a,b; Mavroveli et al., 2009; Petrides D. et al., 2016; Petrides K. V. et al., 2016) offers a comprehensive scientific framework in which to interpret the diverse results of independent empirical research in a way consistent with the longstanding study of individual differences in personality and emotion throughout the lifespan (e.g., Revelle and Scherer, 2009).

The present Research Topic consists of 28 accepted articles on various aspects of Trait EI that stemmed from the collaboration of 97 authors. Each paper reflects rigorous scientific methods to bring together empirical and theoretical research on the construct validity of Trait EI, and to document the most recent advances across the life span. This collection of articles covers a broad scope of subjects, contributed by world-renowned experts in the neurosciences, personality psychology, clinical psychology, developmental psychology, emotions psychology, and education.

## Conceptual Clarification

We agree with Ross Buck that the eventual outcome of emotional education is a greater or lesser degree of Emotional Competence. He defined Emotional Competence for the first time in a scientific paper as “the ability to deal effectively with emotional information -that is, with one's feelings and desires” (Buck, 1990, p. 30, see also Buck, 2014). That same year, Emotional Intelligence (EI) was also presented as a new construct and meticulously analyzed for the first time in a journal article, where it was defined as “the subset of social intelligence that involves the ability to monitor one's own and others' feelings and emotions, to discriminate among them and to use this information to guide one's thinking and actions” (Salovey and Mayer, 1990, p. 189). As it happens, 2020 marks 30 years since the beginnings of both the concepts of Emotional Competence and EI in psychological science.

Emotional Competence has been used as an umbrella term and as a multifaceted construct encompassing a collection of emotion-related skills (e.g., emotion knowledge, emotion perception emotion regulation), abilities, and dispositional qualities (e.g., emotional self-efficacy), that enable an individual to function effectively in emotion-laden situations (Davis and Qualter, 2020). While the concept, or at least the general idea, of Emotional Competence has occasionally been used in literature that is lacking a comprehensive theory or model, EI has attracted unprecedented scientific attention and publications over the last 30 years, and it has become the unifying framework for describing and assessing multiple components of Emotional Competence.

The EI literature has progressively led to “two primary research streams” (Barchard et al., 2016, p. 289), according to which EI can be conceptualized and measured in two distinct, albeit, complementary constructs (Petrides, 2001; Pérez et al., 2005; Stough et al., 2009; Keefer et al., 2018); (1) as a typical performance tested personality trait (Trait EI), representing behavioral dispositions and perceptions of emotion-related competencies (Pérez-González and Sanchez-Ruiz, 2014; Petrides K. V. et al., 2016); or (2) as a maximum performance construct, comprising a set of intellectual abilities for reasoning about emotion (Ability EI; Mayer et al., 2016). Taken together, these two approaches to EI correspond conceptually and operationally with many aspects of Emotional Competence (Davis and Qualter, 2020). Given that Trait EI is conceived as a personality trait and Ability EI as a type of intelligence, Alba-Juez and Pérez-González (2019) suggested that the former might be reinterpreted as the non-cognitive/automatic (temperamental) component of Emotional Competence while the latter might be interpreted as the cognitive/conscious component.

Trait EI is the construct that empirical and meta-analytical evidence has consistently demonstrated to have greater criterion validity (for a summary, see Pérez-González and Qualter, 2018). In fact, Trait EI is consistently positively and significantly related to the most relevant areas of interest for prosperity and happiness in life across the life span, such as well-being, health, romantic and social relationships, leadership, psychosocial adjustment, academic performance, or job performance, and job satisfaction (e.g., Andrei et al., 2016; Keefer et al., 2018; Lea et al., 2018; Di Fabio and Saklofske, 2019; Sarrionandia and Mikolajczak, 2019; Piqueras et al., 2020).

The collection of the papers that constitute this Research Topic clearly support and extend the criterion validity of Trait EI.

## Foundations

A collection of 13 articles expand the nomological network of Trait EI and explore current and new interpretations of the construct or its facets, together constituting a contribution to the foundations of Trait EI theory.

Three papers present new integrative models of EI: Vesely-Maillefer et al. introduce and test a theoretical framework explaining how Trait EI, Ability EI and emotion information processing are related to each other and jointly predict performance in a Theory of Mind task in university students. Agnoli et al. test a model of the combined explanatory role of both Trait EI and fluid non-verbal intelligence in emotional awareness in children, adding to an under-investigated area. Hughes and Evans discuss the Integrated Model of Affect-related Individual Differences (IMAID) as an integrated mediation model in which emotion regulation mediates the effects of ability EI and affect-related personality traits upon outcomes thereby opening a new line of research for future studies focused on testing the validity of this model.

Szczygiel and Mikolajczak (a) show how extraversion predicts greater likeability among adolescents who score high on the interpersonal dimension of Trait EI in contrast to adolescents who score low on this dimension. This demonstrates the moderating role of interpersonal Trait EI in the relationship between extraversion and peer-rated likeability. In a different study, Szczygiel and Mikolajczak (b) demonstrate how Trait EI buffers the deleterious effects of anger and sadness on burnout in a sample of female nurses.

With regard to the importance of Trait EI for health and well-being: Aslanidou et al. examine the Trait EI and general health profiles of parents with and without drug addiction, and also explore the existence of group differences in Trait EI between the offspring of the two groups of parents. Espinosa and Kadić-Maglajlić report a negative relationship between Trait EI and unhealthy behaviors relative to health-promoting ones in a university sample, observing that health consciousness mediated that relationship. Gómez-Baya and Mendoza's study focuses on how trait meta-mood dimensions function as predictors of adaptive response styles to negative affect (i.e., depressive rumination and distraction) and to positive affect (i.e., emotion-focused and self-focused positive rumination and dampening) in adolescents. Sarrionandia et al. present cross-cultural research examining the role of Trait EI as a negative predictor of perceived stress among university students from the United States and Spain through resilience as a mediating variable. Merchán-Clavellino et al. explore the mediating role of trait meta-mood dimensions in the relationship between the motivational BIS/BAS systems (i.e., Behavioral Activation/Approach System and the Behavioral Inhibition System) and affective states of positive and negative affect in university students.

Concerning the relationships between Trait EI and personality, Alegre et al. present a replication study of the Pérez-González and Sanchez-Ruiz's (2014) findings on the inter-relation between Trait EI and personality (i.e., the big five, the two super-factors, and the general factor of personality), using a different trait EI instrument with university students. In a detailed analysis, Fernández-Abascal and Martín-Díaz contribute two studies where they explore the relationships between Trait EI (global and factors scores) and trait meta-mood dimensions with both empathy and non-verbal sensitivity (i.e., people's ability to recognize the communication of feelings, attitudes, and intentions from non-verbal expressions in faces, voice, gestures, and body postures) in university students.

Finally, Smith et al., inspired by computational cognitive neuroscience (CCN), present a novel narrative review of a set of parameters that can be considered candidates for biological markers of different facets of Trait EI. This research has implications for expanding the neural basis of the construct as well as opening a new complementary assessment method to estimate Trait EI through objective measurements.

## Assessment

Since the measurement of EI is a matter of traditional interest in the field, this Research Topic includes four articles focused on instruments for the assessment of the construct or certain of its facets. Austin et al. present two studies on the development and validation of two short forms of the Managing the Emotions of Others (MEOS) Scale, which provides an assessment of the interpersonal emotion regulation facet of Trait EI. Its relationship with global Trait EI, the Big Five, and the dark triad is subsequently explored. Additionally, two articles present the psychometric properties of the Chinese short form (i.e.,) (Feher et al.) and Italian full form (i.e.,) (Chirumbolo et al.) of the Trait Emotional Intelligence Questionnaire (TEIQue) with samples of university students. O'Connor et al. present a comprehensive narrative review of the six major measures of EI in terms of factor structure, reliability, and validity, providing a practical guide with recommendations focused primarily on how to choose between EI constructs (e.g., Ability EI, Trait EI) as well as how to select and use different assessment instruments.

## Education

Finally, 11 articles present direct implications of Trait EI for child and youth development as well as applications in educational settings.

Concerning the role of Trait EI as a predictor of educational and vocational outcomes in youth, three papers make a novel and valuable contribution. From Canada, in a longitudinal investigation, Dave et al. studied a cohort of 1,400 youths at three time points, at age 20–21 years, at age 22–23, and at age 24–25. At each time, they assessed the participants' self-reported educational status, while in the first and third periods they also assessed Trait EI. Their results constitute strong support that higher Trait EI is associated with greater likelihood of pursuit of post-secondary education. From Lebanon, Sanchez-Ruiz and Khouri explore the validity of a model of prediction of academic performance in university students using a novel combination of competing predictors, among which conscientiousness and motivational variables are confirmed as direct predictors, while Trait EI exerts various indirect effects. Farnia et al., using a university sample from United States, investigate the criterion and incremental validity of Trait EI over the Big Five personality traits in career indecisiveness, as well as the role of positive and negative affect mediating this relationship.

Five papers examined how Trait EI contribute to affective processes and mental health. Piqueras et al. test the criterion and incremental validity of Trait EI in the prediction of child psychosocial adjustment, over and above anxiety, depression and sociometric profiles, with particular attention to the role of gender moderating this relationship. In a 1-year longitudinal study, Davis et al. analyze both the separate and combined predictive validity of Ability EI and Trait EI on the maintenance of depressive symptoms and loneliness in middle childhood, also considering the effect of gender. Mestre et al. examine how gender mediates the relationship between emotional states and both dispositional mindfulness and Trait EI in children and adolescents. Foster et al. explored the relationship between dispositional mindfulness, Trait EI, anxiety and depression in adolescents, focusing on how different facets of Trait EI moderate the relation between mindfulness and anxiety and the relation between mindfulness and depression. Using a sample of young adults (i.e., university students), Guil et al. investigate the mediating role of trait meta-mood dimensions in the relationship between self-esteem and trait and state anxiety.

Finally, three papers focused on Trait EI at school. Li et al. study the direct and indirect effects of teachers' Trait EI on self-rated job performance, using a multilevel model where the mediating role of job satisfaction is examined. Two papers test the efficacy of two emotional education programs for adolescents. Rodríguez-Ledo et al., explore the correlations between mindfulness and two questionnaires of Trait EI, an empathy scale, and psychosocial adjustment. They also tested the effectiveness of an emotional education program on some facets of mindfulness. Filella et al. analyze the effectiveness of a gamified emotional education software on Trait EI facets, state anxiety, and academic performance.

## Summary

The collection of articles that comprise this monograph are a testament to the diversity and scientific rigor that is currently characterizing research activity in the study of Emotional Intelligence. At a theoretical level, this special issue demonstrates: (a) the need to integrate and unify emotional intelligence models; (b) the recognition of the prominent place of Trait EI theory in that pursuit; and (c) the promise of elucidating the neuroscientific bases of the construct. On a methodological level, the frequent use of the statistical techniques of moderation/mediation (i.e., 10 out of 26 empirical studies) is a welcome development in order to understand better the mechanisms via which Trait EI relates to other constructs. Regarding measurement, the pre-eminence of the TEIQue is verified as the gold standard for the comprehensive assessment of Trait EI and the operationalization of EC. Finally, on a practical level, it is evident, on the one hand, of the usefulness of considering Trait EI as a key explanatory variable in personal, social, educational and vocational development, and, on the other, the growing diversity of educational approaches for the improvement of Trait EI.

We thank all of the authors who contributed their research to this special issue. The quality and diversity of these research investigations have added significantly to our theoretical and empirical descriptions of EI but have also provided direction for the continuing development of the huge potential that EI has to offer. In this regard, we hope that this special edition of research papers will inspire and provide the basis for future studies that will push forward the frontiers of our knowledge on how to be more emotionally competent. Such contributions can help us move closer to the global goals for sustainable development championed by the United Nations, such as good health and well-being, quality education, decent work and economic growth, and peace, justice and strong institutions.

## Author Contributions

J-CP-G, DS, and SM: conceptualization and writing—review and editing. J-CP-G: writing—original draft preparation. All authors have read and agreed to the published version of the manuscript.

### Conflict of Interest

The authors declare that the research was conducted in the absence of any commercial or financial relationships that could be construed as a potential conflict of interest.

## References

[B1] Alba-Juez L. Pérez-González J.-C. (2019). Emotion and language ‘at work’: the relationship between trait emotional intelligence and communicative competence as manifested at the workplace, in Emotion in Discourse, eds Lachlan Mackenzie J. Alba-Juez L., (Amsterdam: John Benjamins), 247–278. 10.1075/pbns.302.10alb

[B2] Andrei F Siegling A. B Aloe A. M. Baldaro B. Petrides K. V. (2016). The incremental validity of the trait emotional intelligence questionnaire (TEIQue): a systematic review and meta-analysis. J. Personality Assessment 98, 261–276. 10.1080/00223891.2015.1084630 26457443

[B3] Barchard K. A. Brackett M. A. Mestre J. M. (2016). Taking stock and moving forward: 25 years of emotional intelligence research. Emotion Review 8:289 10.1177/1754073916650562

[B4] Buck R. (1990). Rapport, emotional education, and emotional competence. Psychological Inquiry 1, 301–302. 10.1207/s15327965pli0104_4

[B5] Buck R. (2014). Emotion. A Biosocial Synthesis. Cambridge, UK: Cambridge University Press.

[B6] Davis S. K. Qualter P. (2020). Emotional competence. The Encyclopedia of Child and Adolescent Development, eds Hupp S. Jewell J. (Wiley). 10.1002/9781119171492.wecad475

[B7] Di Fabio A. Saklofske D. H. (2019). Positive relational management for sustainable development: Beyond personality traits-the contribution of emotional intelligence. Sustainability 11:330 10.3390/su11020330

[B8] Keefer K. Parker J. D. A. Saklofske D. H. (eds). (2018). Emotional Intelligence in Education: Integrating Research With Practice. New York, NY: Springer.

[B9] Lea R. G. Qualter P. Davis S. K. Pérez-González J. C. Bangee M. (2018). Trait emotional intelligence and attentional bias for positive emotion: an eye tracking study. Pers. Individ. Differ. 128, 88–93. 10.1016/j.paid.2018.02.017

[B10] Mavroveli S. Petrides K. V. Sangareau Y. Furnham A. (2009). Exploring the relationships between trait emotional intelligence and objective socio-emotional outcomes in childhood. Br. J. Educ. Psychol. 79, 259–272. 10.1348/000709908X368848 18950549

[B11] Mayer J. D. Caruso D. R. Salovey P. (2016). The ability model of emotional intelligence: Principles and updates. Emotion Review 8, 290–300. 10.1177/1754073916639667

[B12] Pérez J. C. Petrides K. V. Furnham A. (2005). Measuring trait emotional intelligence, in Emotional Intelligence An International Handbook, eds Schulze R. Roberts R. D. (Cambridge, MA: Hogrefe & Huber), 181–201.

[B13] Pérez-González J-C. Qualter P. (2018). Emotional intelligence and emotional education in school years. An Introduction to Emotional Intelligence, eds Dacree Pool L. Qualter P. (Chichester: Wiley), 81–104.

[B14] Pérez-González J. C. Sanchez-Ruiz M. J. (2014). Trait emotional intelligence anchored within the big five, big two and big one frameworks. Personality and Individual Differences 65, 53–58. 10.1016/j.paid.2014.01.021

[B15] Petrides D. Siegling A. Saklofske D. H. (2016). Theory and measurement of trait emotional intelligence. Wiley Handbook of Personality Assessment, ed Kumar U. (John Wiley), 90–103.

[B16] Petrides K. V. (2001). A psychometric investigation into the construct of emotional intelligence (Doctoral dissertation). University College London.

[B17] Petrides K. V. Mikolajczak M. Mavroveli S. Sanchez-Ruiz M. J. M. J. Furnham A. Pérez-González J. C. (2016). Developments in trait emotional intelligence research. Emotion Review, 8, 335–341. 10.1177/1754073916650493

[B18] Petrides K. V. Pérez-González J. C. Furnham A. (2007a). On the criterion and incremental validity of trait emotional intelligence. Cogn. Emot. 21, 26–55. 10.1080/02699930601038912

[B19] Petrides K. V. Pita R. Kokkinaki F. (2007b). The location of trait emotional intelligence in personality factor space. Br. J. Psychol. 98, 273–289. 10.1348/000712606X120618 17456273

[B20] Petrides K. V. Sanchez-Ruiz M. J. Siegling A. B. Saklofske D. H. Mavroveli S. (2018). Emotional intelligence as personality: measurement and role of trait emotional intelligence in educational contexts. Emotional Intelligence in Education. Integrating Research With Practice, eds Keefer K. V. Parker J. D. A. Saklofske D. H. (Cham: Springer) 49–81.

[B21] Piqueras J. A. Salvador M. D. C. Soto-Sanz V. Mira F. Pérez-González J. C. (2020). Strengths against psychopathology in adolescents: ratifying the robust buffer role of trait emotional intelligence. International Journal of Environmental Research and Public Health 17:804. 10.3390/ijerph17030804 32012879 PMC7037399

[B22] Revelle W. Scherer K. R. (2009). Personality and emotion. Oxford Companion to Emotion and the Affective Sciences, eds Sander D. Scherer K. R. (Oxford, UK: Oxford University Press), 21–28.

[B23] Salovey P. Mayer J. D. (1990). Emotional intelligence. Imagination, Cognition And Personality 9, 185–211. 10.2190/DUGG-P24E-52WK-6CDG

[B24] Sarrionandia A. Mikolajczak M. (2019). A meta-analysis of the possible behavioural and biological variables linking trait emotional intelligence to health. Health Psychology Review. 10.1080/17437199.2019.1641423 31284846

[B25] Stough C. Saklofske D. H. Parker J. (eds.). (2009). Assessing Emotional Intelligence: Theory, Research and Applications. New York, NY: Springer.

